# Supplementary material for: A systematic review and Bayesian meta-analysis provide evidence for an effect of acute physical activity on cognition in young adults
Source: Commun Psychol. 2024 Aug 28;2:82. doi: 10.1038/s44271-024-00124-2 (PMC11358546; doi:10.1038/s44271-024-00124-2)
Supplement: Supplementary file 2 — Supplementary Material [file 44271_2024_124_MOESM2_ESM.pdf]

Supplementary Table 1. Cognitive task classification criteria.

Cognitive Task	Classified Domain	EF Sub-Domain
0-back	Attention	-
Attention Networks Test (Overall & Alerting)	Attention	-
Connors/ Rosvold Continuous Performance Test	Attention	-
Continual Processing Task	Attention	-
Continuous Monitoring Task	Attention	-
Continuous Visual Attention Task	Attention	-
Digit Symbol Coding	Attention	-
Digital Finger Tapping Test	Attention	-
Divided Attention Task	Attention	-
Dot-Probe Task	Attention	-
Global vs Local Detection	Attention	-
Go/No-Go Task (Redundant Target)	Attention	-
Harris Concentration Grid Test	Attention	-
Internal Shift Task	Attention	-
Landolt C Test	Attention	-
Multitasking Test	Attention	-
Oddball Task	Attention	-
Paced Auditory Serial Addition Test	Attention	-
Perception and Attention Function battery	Attention	-
Posner Cueing Task	Attention	-
Pro-Response Task	Attention	-
Spatial Cueing	Attention	-
Stroop Task (Congruent)	Attention	-
Sustained Attention to Response Task	Attention	-
Trail Making Test A	Attention	-
Vienna Test Task	Attention	-
Viral Video Prospective Memory (detection)	Attention	-
Visual Search	Attention	-
Visual Threat Detection	Attention	-
Visual Vigilance	Attention	-
Anti-Response Task	Executive Function	Cognitive Control
Digit Symbol Substitution	Executive Function	Cognitive Control
Task Switching Paradigm	Executive Function	Cognitive Control
Trail Making B	Executive Function	Cognitive Control
Decision and Shoot Task	Executive Function	Decision Making
Logical Reasoning Task	Executive Function	Decision Making
Naval Choice RT Task	Executive Function	Decision Making
Soccer specific decision making task	Executive Function	Decision Making
Speed-discrimination task	Executive Function	Decision Making
Angram Task	Executive Function	Flexibility

Dimension Switching Task	Executive Function	Flexibility
Anti-saccade Task	Executive Function	Inhibition
Flanker Task	Executive Function	Inhibition
Go/No-Go + Flanker Task	Executive Function	Inhibition
Go/No-Go Task	Executive Function	Inhibition
Simon Task	Executive Function	Inhibition
Simple/Complex Visual Discrimination Task	Executive Function	Inhibition
Stroop Task (Incongruent/Interference)	Executive Function	Inhibition
Controlled Oral Word Association Test	Executive Function	Language
Ruff Figural Fluency Task	Executive Function	Language
Tower of London	Executive Function	Planning
N-back (N > 0)	Executive Function	Working Memory
Auditory Consonant Trigram Test	Executive Function	Working Memory
Brown-Peterson Test	Executive Function	Working Memory
Code Substitution	Executive Function	Working Memory
Deese-Roediger-McDermott Paradigm (Recall immediately)	Executive Function	Working Memory
Delayed matching-to-sample task	Executive Function	Working Memory
Digit Span	Executive Function	Working Memory
Letter Number Sequencing	Executive Function	Working Memory
Math Problems Task	Executive Function	Working Memory
Non-Verbal Memory Test	Executive Function	Working Memory
Operation Span Task	Executive Function	Working Memory
Phoneme Monitoring	Executive Function	Working Memory
Plus-Minus Task	Executive Function	Working Memory
Self-Ordered Pointing Test	Executive Function	Working Memory
Serial addition/subtraction	Executive Function	Working Memory
Spatial Delayed Response Task	Executive Function	Working Memory
Spatial Span Task	Executive Function	Working Memory
Sternberg	Executive Function	Working Memory
Verbal Runing Span	Executive Function	Working Memory
Visual Spatial Memory	Executive Function	Working Memory
Auditory Choice RT	Information Processing	-
Choice RT Task	Information Processing	-
CogState Battery Choice RT Task	Information Processing	-
CogState Battery Detection Task	Information Processing	-
Simple RT Task	Information Processing	-
Repeated Acquisition	Learning	-
Embedded Triplet Learning Task	Memory	-

Implicit Vocabulary Learning Task	Memory	-
Motor Skill Acquisition	Memory	-
Multiple Choice Test	Memory	-
Auditory Tone Familiarity	Memory	-
Brief Visuospatial Memory Test-Revised	Memory	-
California Verbal Learning Test II	Memory	-
Code Substitution Delayed	Memory	-
Customized Online Vocabulary Test	Memory	-
Deese-Roediger-McDermott Paradigm (Recall 25 min post)	Memory	-
Recall Tasks	Memory	-
Recognition Tasks	Memory	-
Rey Auditory Verbal Learning Test	Memory	-
Soccer specific decision making task	Memory	-
Word Association	Memory	-
Kicking Accuracy Test	Motor Skills	-
Rifle Shooting	Motor Skills	-
Shooting Accuracy	Motor Skills	-
Luminance Discrimination Task	Perception	-
Critical Flicker Fusion	Perception	-
Detection Task	Perception	-
Hybrid Flanker/Load Task	Perception	-
Simple Visual-discrimination task	Perception	-
Visual Consciousness	Perception	-
